# Protein change in plant evolution: tracing one thread connecting molecular and phenotypic diversity

**DOI:** 10.3389/fpls.2013.00382

**Published:** 2013-10-10

**Authors:** Madelaine E. Bartlett, Clinton J. Whipple

**Affiliations:** Biology Department, Brigham Young UniversityProvo, UT, USA

**Keywords:** molecular evolution, structural mutations, protein evolution, coding vs. non-coding changes, plant evo-devo, genotype to phenotype map

## Abstract

Proteins change over the course of evolutionary time. New protein-coding genes and gene families emerge and diversify, ultimately affecting an organism’s phenotype and interactions with its environment. Here we survey the range of structural protein change observed in plants and review the role these changes have had in the evolution of plant form and function. Verified examples tying evolutionary change in protein structure to phenotypic change remain scarce. We will review the existing examples, as well as draw from investigations into domestication, and quantitative trait locus (QTL) cloning studies searching for the molecular underpinnings of natural variation. The evolutionary significance of many cloned QTL has not been assessed, but all the examples identified so far have begun to reveal the extent of protein structural diversity tolerated in natural systems. This molecular (and phenotypic) diversity could come to represent part of natural selection’s source material in the adaptive evolution of novel traits. Protein structure and function can change in many distinct ways, but the changes we identified in studies of natural diversity and protein evolution were predicted to fall primarily into one of six categories: altered active and binding sites; altered protein–protein interactions; altered domain content; altered activity as an activator or repressor; altered protein stability; and hypomorphic and hypermorphic alleles. There was also variability in the evolutionary scale at which particular changes were observed. Some changes were detected at both micro- and macroevolutionary timescales, while others were observed primarily at deep or shallow phylogenetic levels. This variation might be used to determine the trajectory of future investigations in structural molecular evolution.

## INTRODUCTION

In the study of the molecular changes underlying adaptive evolution, there is debate as to whether regulatory or structural changes are of greater importance. Regulatory changes, especially those affecting where and when a transcriptional regulator is expressed, are thought to predominate. Structural changes are thought to have a higher degree of negative pleiotropy, and are probably not tolerated to the same degree as regulatory changes (Carroll, 2000, 2005, 2008; Stern, 2000). Despite this prevailing view, structural changes have been shown to have had a noteworthy role in the evolution of some key adaptive traits (Hoekstra and Coyne, 2007). In the evolution of plant form and function in particular, examples of both regulatory (Arnaud et al., 2011) and structural (Airoldi et al., 2010) changes exist. With time and more data, the argument may be resolved, but the point that every trait is different may be key (Wessinger and Rausher, 2012). In all likelihood, in most cases there is no single quantitative trait nucleotide (QTN), but rather a collection of myriad small changes, both regulatory and structural, that have contributed to the evolution of a novel phenotype (Rockman, 2012).

Regardless of which class of changes predominates, both regulatory and structural mutations have happened through the course of evolution. We have chosen to review those cases where structural mutations have had demonstrably functional consequences. Interpreting a mutation as either regulatory or structural is not always straightforward. We use the definition proposed by Hoekstra and Coyne (2007), with some modifications. They propose that mutations that occur in the coding sequences of genes are structural, and all other mutations, including those that occur in introns, are regulatory. This definition includes nonsense null mutations, altered miRNA-binding sites, and silent mutations affecting transcription and translation dynamics as “structural” (Hoekstra and Coyne, 2007). We prefer a more narrow definition of “structural mutation,” and include only those examples where amino acid sequence is changed and protein function is not completely lost. This circumscription of structural mutations thus includes mostly missense mutations, but also insertions and deletions, frameshifts, and premature stop codons that produce proteins with altered functions. We have chosen this definition out of expediency. Compelling arguments exist for putting all excluded mutations (e.g., miRNA-binding site mutations) back into the set of structural mutations, and then for taking them right back out again. For the purpose of investigating the evolution of protein function, we feel that our narrow definition best frames the discussion.

Our review focuses on those cases where protein function has been altered, in turn altering some aspect of phenotype. It is important to highlight that many of the phenotypes we discuss may or may not be adaptive, but that is not the focus of this review. It is not trivial to unambiguously determine the molecular cause of a phenotypic adaptation, or even to confirm some phenotype as adaptive (Barrett and Hoekstra, 2011). Moreover, there are few studies that have explicitly investigated the quantitative trait loci (QTL) underlying natural variation in an evolutionary framework, and as a consequence it is hard to determine their adaptive significance. A more widespread genotype might hint at some adaptive value, but for many of the examples we cite the responsible protein change is only found in a single accession where it may be deleterious and/or of short duration. These isolated QTL are not inherently less interesting, however, because they reveal the scope of molecular diversity to be found in natural environments, diversity that selection may ultimately act upon.

Another important preliminary consideration is that protein diversification through deep time can only be discussed in a framework of gene birth. In plants in particular, and in eukaryotes in general, a major source of new genes is gene duplication. Most gene families have expanded considerably through gene duplication and divergence, and often these expansions show lineage-specific patterns (Flagel and Wendel, 2009). The new gene duplicates are thought to have one of three fates. Formally, duplicate genes may divide up the functions of the progenitor gene between them (subfunctionalization), one of the duplicates may gain an entirely new function (neofunctionalization), or one of the duplicates may decay into a non-functional pseudogene (Ohno, 1970; Lynch et al., 2001). These categories are often difficult to assign, but where they are relevant, most of the examples we will discuss are of neofunctionalization.

**FIGURE 1:**
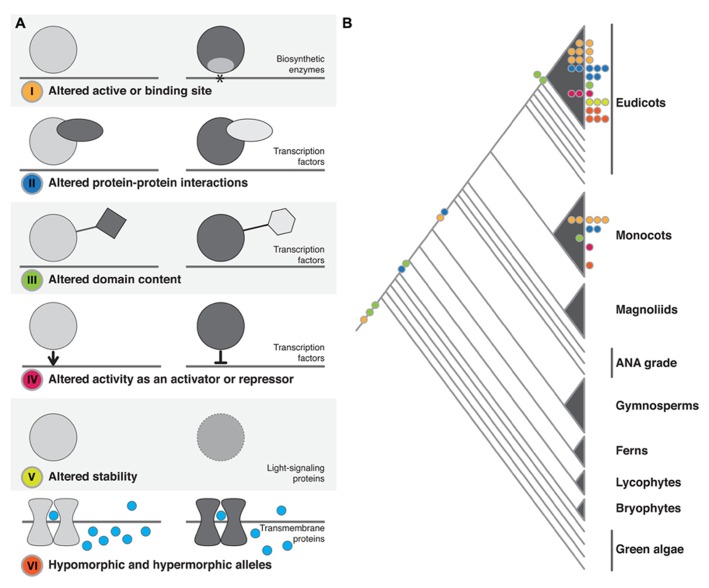
**Structural changes observed in plant phenotypic variation and evolution.** **(A)** The six classes of structural change identified. The dominant family of proteins identified as being affected by each class of change is noted. In I–IV the gray line represents DNA, in VI it represents the cell membrane. **(B)** The approximate phylogenetic placement of described structural changes. Colored circles on branches or within clades represent change at the macroevolutionary level. Colored circles at tips represent microevolutionary changes (color coding as in **A**).

In our review of the literature, we found that functional protein changes, the result of underlying structural mutations, fell into six broad, non-mutually exclusive categories. We have divided up our discussion according to these categories: (I) altered active or binding sites; (II) altered protein–protein interactions; (III) altered domain content; (IV) altered activity as a transcriptional activator or repressor; (V) altered protein stability; or (VI) hypomorphic and hypermorphic alleles (**Figure 1** and **Table 1**).

**Table 1:** Structural changes implicated in phenotypic variation and evolution in plants.

Organism	Protein	Trait	Amino acid change	Scale^*^	Reference
**Altered active or binding site**
*Arabidopsis thaliana*	Cytochrome P450’s	Arabidopyrone synthesis	Gradual replacement	M	Weng et al. (2012)
*Boechera stricta*	CYP79F1 duplicates	Glucosinolate biosynthesis	G134L, P536K	M	Prasad et al. (2012)
*Eleusine indica*	α-tubulin	Herbicide resistance	T239I	m	Yamamoto et al. (1998)
*Ipomoea spp.*	Dfr	Flower color	Five amino acid sites	M	Smith et al. (2013)
*Oryza sativa*	SH4	Seed abscission	K80N, DNA-binding domain	m	Li et al. (2006)
*Setaria viridis*	α-tubulin	Herbicide resistance	T239I	m	Anthony et al. (1998)
*Solanum lycopersicum*	LIN5	Sucrose metabolism	E348D, close proximity to catalytic site	m	Fridman et al. (2004)
Asteraceae	HHS	Pyrrolizidine alkaloid synthesis	Gradual replacement	M	Anke et al. (2004)
Convolvulaceae	HHS	Pyrrolizidine alkaloid synthesis	Gradual replacement	M	Kaltenegger et al. (2013)
Flowering plants	psbA	Herbicide resistance	S264G	m	Powles and Yu (2010)
Flowering plants	AHAS	Herbicide resistance	Seven amino acid sites	m	Powles and Yu (2010)
Land plants	LEAFY	Vegetative to reproductive transition	Gradual replacement	M	Maizel et al. (2005)
Eukaryotes	MADS TF’s	Morphogenesis	Gradual replacement	M	Gramzow et al. (2010)
**Altered protein–protein interactions **
*Antirrhinum majus*	FAR, PLE	Floral organ identity	Q insertion (K-C domain junction)	M	Airoldi et al. (2010)
*Arabidopsis thaliana*	ATMYC1	Trichome density	P189A	m	Symonds et al. (2011)
*Arabidopsis thaliana* (Cvi)	PHYB	Light response	I143L (PPI domain)	m	Filiault et al. (2008)
*Arabidopsis thaliana*	AGL6	Branching	P201L (C-terminal)	m	Huang et al. (2012)
*Helianthus annuus*	FT	Flowering time	Frameshift mutation, 17aa insertion	m	Blackman et al. (2010)
*Hordeum vulgare*	PPD-H1	Flowering time	G588W in the CCT domain	m	Turner et al. (2005)
*Medicago*	SHP homologs	Fruit morphology	S insertion (C-terminal)	M	Fourquin et al. (2013)
*Thalictrum thalictroides*	C class MADS TF	Floral organ identity	8 or 13 amino acid deletion, Keratin-like domain	m	Galimba et al. (2012)
*Triticum aestivum*	*Q*	Seed free-threshing	I329V	m	Simons et al. (2006)
Land plants	DELLA	GA-mediated growth responses	Gradual changes to DELLA, affecting DELLA-GID1 PPI	M	Yasumura et al. (2007)
Flowering plants	B class MADS TF	Floral development	Uncharacterized	M	Winter et al. (2002)
**Altered domain content **
*Arabidopsis thaliana* (Cvi)	ANAC089	Fructose sensitivity	Truncated protein, membrane-anchoring domain lost.	m	Li et al. (2011)
Core eudicots	EuAP3 and TM6	Floral development	Frameshift mutation, novel C-terminal	M	Kramer et al. (2006)
Core eudicots	EuAP1 and EuFUL	Floral development	Frameshift mutation, novel C-terminal	M	Litt and Irish (2003); Pabón-Mora et al. (2012)
Grasses* (Oryza sativa)*	OsMADS5	Floral morphology	Truncated protein, C-terminal lost	M	Christensen and Malcomber (2012)
Land plants	Terpene synthases	Secondary metabolism	γ-domain lost	M	Hillwig et al. (2011)
Green plants	AP2 domain	Development, stress response	HGT of novel domain	M	Magnani et al. (2004)
Green plants	MEKHLA domain	Development	HGT of novel domain	M	Mukherjee and Buerglin (2006)
**Altered activity as an activator or repressor **
*Beta vulgaris*	BvFT1, BvFT2	Flowering, vernalization	Three amino acids in segment B	M	Pin et al. (2010)
*Glycine max*	Ln	Leaf morphology	D9H, EAR transcriptional repression motif	m	Jeong et al. (2012)
*Zea mays*	TGA1	Inflorescence morphology	K8N	m	Wang et al. (2005)
Angiosperms *(Arabidopsis thaliana)*	TFL, FT	Flowering	Y85H (FT to TFL), H88Y (TFL to FT)	M	Hanzawa et al. (2005)
**Altered protein stability **
*Arabidopsis thaliana *(Lm-2)	PHYA	Light response	M548T	m	Maloof et al. (2001)
*Arabidopsis thaliana* (Cvi)	CRY2	Light response	V367M	m	El-Assal et al. (2001)
*Arabidopsis thaliana*	ETC2	Trichome density	K19E	m	Hilscher et al. (2009)
**Hypomorphic and hypermorphic alleles**
*Arabidopsis thaliana* (Cvi)	HMA5	Cu tolerance	Missense mutations in conserved transmembrane domain	m	Kobayashi et al. (2008)
*Arabidopsis thaliana*	HMA3	Cd accumulation	Missense mutations at ATP-binding site (or nearby)	m	Chao et al. (2012)
*Arabidopsis thaliana* (Shahdara)	APR2	Sulfate accumulation	A399E	m	Loudet et al. (2007)
*Arabidopsis thaliana*	*ACD6*	Late onset necrosis, leaf initiation	Two or three aa replacements in transmembrane domain	m	Todesco et al. (2010)
*Arabidopsis thaliana* (Sy-0)	*HUA2*	Plant architecture	K525E	m	Wang et al. (2007)
*Oryza sativa*	SKC1	Salt tolerance	Four amino acids, transmembrane domain	m	Ren et al. (2005)
**Uncertain function **
*Arabidopsis lyrata*	FRI-like	Flowering	14 aa insertion	m	Kuittinen et al. (2008)
*Oryza sativa*	PROG1	Plant architecture (tillering)	T152S	m	Jin et al. (2008)

* M = macroevolutionary scale, m = microevolutionary scale.

## ALTERED ACTIVE AND BINDING SITES

Amino acid replacement in the active sites of enzymes, or the DNA-binding sites of transcription factors, is perhaps the most easily understood mechanism of protein evolution. Changes to the core functional domain of a protein, either through gradual replacement of many amino acids over the course of time (Zhao et al., 2008), or through the replacement of a few key residues (Greenhagen et al., 2006; O’Maille et al., 2008), has the potential to generate novel protein function. Active and binding site changes also have the greatest potential to be deleterious if they destroy a protein’s primary function (Carroll, 2008). Despite this potential for negative effects, numerous examples (outlined below) have been uncovered where active and binding site evolution has been tolerated and led to neofunctionalization.

### SECONDARY METABOLITES IN DEFENSE

Plants are remarkable for their secondary metabolite chemistry: they possess a diversity of chemical compounds, often involved in defense (Dixon, 2001). Gene duplication followed by neofunctionalization is a novelty-generating mechanism observed frequently in the evolution of enzymes and secondary metabolites. Gene duplication followed by active site evolution has been described in the synthesis of the arabidopyrones (*Arabidopsis*-specific compounds; Weng et al., 2012); glucosinolates in the *Arabidopsis *relative *Boechera *(Prasad et al., 2012); and pyrrolizidine alkaloids in the Convolvulaceae and the Asteraceae (Anke et al., 2004; Reimann et al., 2004). Both in the evolution of novel glucosinolate-producing enzymes in *Boechera *(Prasad et al., 2012), and in the evolution of pyrrolizidine alkaloid production in the Convolvulaceae (Kaltenegger et al., 2013), positive selection acting on active site amino acid residues was detected. The positively selected residues were assayed for function, and found to indeed alter enzyme function in predictable ways (Prasad et al., 2012; Kaltenegger et al., 2013). This pattern of gene duplication, positive selection, and neofunctionalization has been proposed as a mechanism for glucosinolate biosynthesis evolution in the Brassicaceae (Benderoth et al., 2006), and appears to be relevant for a broader spectrum of secondary metabolite evolution.

A second theme observed in the evolution of novel enzymes is that of a promiscuous enzyme becoming more specialized through the course of evolution. In both pyrrolizidine alkaloid and arabidopyrone synthesis, the enzyme maintaining ancestral function shows weak activity toward the substrate used by the neofunctionalized enzyme (Weng et al., 2012; Kaltenegger et al., 2013). In these cases, which may be fairly prevalent, the catalytic activity of the progenitor enzyme may be considered a molecular exaptation. An exaptation, as defined by Gould and Vrba (1982), is a feature coopted for some current function following an origin for a different function, or no function at all. Promiscuous catalytic activity of an enzyme may serve as an exaptation in the evolution of new enzyme functions after gene duplication (O’Brien and Herschlag, 1999; Aharoni et al., 2004). This may also still be considered neofunctionalization, depending on the definition of function used. If an evolutionary definition of function is used – an enzyme’s function is the function it was selected for – then the increased specialization is indeed neofunctionalization. If, instead, we choose a purely mechanical definition – a promiscuous enzyme functions to produce a range of products – then exaptation, but not neofunctionalization, would be better applied. In the case of biochemical enzymes, many neofunctionalization events may be exaptations, but not all neofunctionalization events are because of exaptation.

### HERBICIDE RESISTANCE

Herbicide resistance, both naturally and experimentally derived, is often the result of structural changes, particularly in the active sites of enzymes. The possible shifts to resistance in an herbicide-sensitive protein is dependent on where a particular herbicide binds. If an herbicide binds within an enzyme’s catalytic site, there are relatively few amino acid changes that can confer resistance, while still maintaining catalytic activity. If an herbicide binds outside of an enzyme’s catalytic site, a larger spectrum of changes can confer resistance while still maintaining enzyme function. Because herbicide treatment represents extremely strong selective pressure, applied in agricultural settings worldwide, and because both sets of tolerated amino acid changes are relatively small, the evolution of herbicide resistance is a story of molecular convergence. For example, a single amino acid change that confers triazine herbicide resistance in a key photosystem II gene, *psbA* (S264G), has evolved independently at least 68 times worldwide. Similarly, 22 amino acid replacements at seven sites in the enzyme acetohydroxyacid synthase (AHAS) have been identified in herbicide-resistant weeds (reviewed in Powles and Yu, 2010). In a final example of molecular convergence, the same herbicide resistance-conferring mutation (T239I) has arisen separately in the α-tubulin genes of the grasses *Eleusine indica* and *Setaria viridis *(Anthony et al., 1998; Yamamoto et al., 1998).

### FLOWER COLOR EVOLUTION

Flower color evolution is another domain where structural changes in enzymes, along with regulatory changes and enzyme inactivations, have been shown to be important (Wessinger and Rausher, 2012). In *Iochroma* (Solanaceae) a color change from blue (ancestral) to red (derived) occurred because of three changes: inactivation of one enzyme, downregulation of a second by a distinct locus, and altered functional specificity of a third (Dfr; Smith and Rausher, 2011). It remains unclear which changes occurred first, and were ultimately responsible for the color shift, but it *is* clear that changes in Dfr specificity occurred both before and after the emergence of the red-flowered ancestor. The five amino acids that differ between the red-flowered and blue-flowered ancestral proteins evolved under positive selection. Ancestral sequence estimation, coupled to site-directed mutagenesis and functional assays revealed that each amino acid change, when it occurs in a specific protein sequence background, confers progressively more specificity for the red color precursor. These results suggest that each of the amino acid changes in Dfr may have been adaptive (Smith et al., 2013).

### DNA-BINDING SITE EVOLUTION

Regulatory changes are often considered more prevalent in the evolution of transcription factor function, and hence in the evolution of morphology. However, there is evidence that binding (active) site evolution is of some importance in the evolution of the LEAFY (LFY) and MADS box transcription factors. The *A. thaliana *protein LFY, like its orthologs in other flowering plants, is a floral integrator and a master regulator of floral organ identity (Moyroud et al., 2010). In the moss *Physcomitrella patens*, however, the two *LFY *genes control the first zygotic cell division and numerous aspects of sporophyte development, not the vegetative to reproductive transition in the sporophyte (Tanahashi et al., 2005). In *Ceratopteris*, a fern, the expression patterns of *LFY* homologs and other MADS box genes are not overlapping, suggesting that LFY does not induce MADS box gene expression, as it does in the flowering plants (Himi et al., 2001). Changes in the DNA-binding domain appear to have been important in this altered functional specificity of LFY across the evolutionary history of land plants. Heterologous expression studies, domain swaps, and site-directed mutagenesis experiments suggest that gradual amino acid replacement in the DNA-binding domain, through the course of plant evolution, may have been of some importance in the evolution of altered LFY function (Maizel et al., 2005).

The MADS box genes are found in almost all eukaryotic genomes, and have expanded considerably in plant genomes in particular. Plant MADS box genes have key roles in many morphogenetic processes, including flowering, floral development, and fruit development. Careful and exhaustive database searches and phylogenetic analyses have revealed that the MADS box genes of eukaryotes may have evolved from a gene encoding a topoisomerase subunit (TopoIIA subunit A). DNA topoisomerases, like TopoIIA, have central roles in DNA replication, transcription, recombination, and chromosome segregation. Gradual changes in the DNA-binding domain may have eventually led to the DNA-binding specificity for CArG boxes observed in MADS box proteins (Gramzow et al., 2010).

A single amino acid replacement (K80N) in the MYB domain transcription factor SHATTERING4 (SH4) is responsible for the non-shattering phenotype characteristic of cultivated rice (Li et al., 2006; Zhu et al., 2012). In wild rice species, the seeds abscise from the inflorescence axis (shattering) because of the formation of an abscission zone. In cultivated rice, seeds are retained on the inflorescence axis and the abscission zone is reduced, allowing for easier harvest. K80N is in the DNA-binding domain of sh4 and probably undermines or changes (but not abolishes) protein function, thus interrupting abscission layer formation (Li et al., 2006).

Structural active site changes may well be tolerated at a higher frequency in biosynthetic enzymes, and lead to novel phenotypes more often than analogous changes in transcription factors, but we see no particular reason to consider the evolution of transcription factors and the evolution of biochemical enzymes as two fundamentally distinct processes. We suspect that one of the recurrent themes identified in enzyme evolution – gene duplication followed by neofunctionalization – may rather become a more general theme in protein evolution. Gradual binding site evolution of transcription factors, as demonstrated in LFY and suggested in the MADS box proteins, may be more widespread. Although DNA-binding domains are often deeply conserved in gene families, it remains conceivable that the DNA-binding profile of a transcription factor may diverge following a gene duplication event. It is fairly laborious to identify transcription factor binding sites, even in model organisms. More sequenced genomes, however, along with new techniques such as chromatin immunoprecipitation coupled to next generation sequencing (ChIP-Seq) may allow us to uncover more examples of structural transcription factor evolution. ChIP-Seq has the potential to reveal altered DNA-binding profiles through time, whether this is because of altered binding sites, altered protein–protein interactions (PPIs), or other mechanisms. This is not to discount the demonstrated importance of changes in transcription factor gene expression in morphological evolution (Arnaud et al., 2011), but only to highlight the potential importance of structural and regulatory changes occurring together through deep time.

Molecular convergence may also become a more general theme in protein evolution (Gherardini et al., 2007). As with biosynthetic enzymes, a protein with DNA-binding activity has a finite genotypic space to explore in adopting some new function (binding a new DNA motif, for example) (Wagner, 2011). Consequently, the subset of changes that can occur at key residues is relatively small. Further examples may reveal recurrent changes in homologous protein domains not just in biosynthetic enzymes and herbicide-targeted proteins, but also in transcription factors.

## ALTERED PROTEIN–PROTEIN INTERACTIONS

Protein–protein interactions are of prime importance in plant development. There are many examples of particular interactions regulating key developmental and physiological processes (e.g., Riechmann et al., 1996a; Kim et al., 1997; Cui et al., 2007). Altered PPIs may be one way to generate functional diversity without negatively affecting core protein function. The DNA-binding domain of a protein may stay intact, but an interaction domain may change to interact with a new partner, perhaps expressed in a discrete domain. In this way novel functions can emerge, while the protein’s original functions are preserved (Lynch and Wagner, 2008). Despite this compelling argument for investigating PPI evolution, and despite their integral role in development, few studies have tackled PPIs in an evolutionary framework.

One interaction that has been studied in an evolutionary context occurs between the gibberellin phytohormones (GA), GID1-like proteins (GLP1), and the DELLA transcriptional repressors. In *A. thaliana* DELLA proteins, as part of GLP1–GA–DELLA complexes, are polyubiquitinated and recruited to the 26S proteasome for destruction, releasing DELLA targets from repression (reviewed in Sun, 2011). The GLP1–GA–DELLA interaction is deeply conserved in angiosperms (Sun, 2011), and appears to have been acquired gradually through the course of land plant evolution (Yasumura et al., 2007). The results of mutant analyses and heterologous transformation experiments suggest that DELLA’s acquired their characteristic growth-repressive function after the divergence of the lycophytes from the rest of the land plants, perhaps through *cis*-regulatory changes. The GA-stimulated GLP1–DELLA interaction appears to have arisen after the divergence of the bryophytes from the remainder of the land plants, probably through structural alterations to DELLA proteins. Thus DELLA protein changes that facilitate the GLP1–DELLA interaction, together with the evolution of an altered GA response, allowed for the emergence of the GLP1–GA–DELLA module characteristic of flowering plants (Yasumura et al., 2007).

In the study of plant development, the network of interactions between the ABC(E) MADS box proteins has been extensively investigated. The ABC(E) class MADS box genes, and the single non-MADS A class gene *AP2* (APETALA2), control floral organ identity in a combinatorial manner. In *Arabidopsis* and *Antirrhinum* the A class genes control sepal identity. The A and B class genes together control petal identity, B and C class genes together confer stamen identity, and the C class genes control carpel identity (Coen and Meyerowitz, 1991). The E class genes are needed in all four whorls of the flower for proper organ identity specification (Pelaz et al., 2000; Honma and Goto, 2001). The ABC(E) MADS box proteins are known to dimerize, but probably function as part of tetramers (“floral quartets”). These proteins have four domains: the DNA-binding MADS domain, an Intervening domain (I), a keratin-like coiled coil (K), and a disordered C-terminal domain. The I, K, and C-domains have been implicated in mediating PPIs amongst MADS box proteins (reviewed in Immink et al., 2010).

There are a few examples where novel mutant phenotypes are probably caused by disrupted PPIs of MADS box transcription factors. The fast neutron induced *seirena* mutant of the California poppy, *Eschscholzia californica* (Ranunculaceae), shows a B class mutant phenotype, and may result from compromised interactions between the B class, C class, and E class MADS box proteins. Site-directed mutagenesis experiments revealed that the B–C–E interaction in *Eschscholzia *may be mediated by the PISTILLATA (PI) motif, missing from sei-1 mutant protein (Lange et al., 2013). The PI motif is conserved, but not universally present, in PI homologs. Although the PI motif may well have a role in MADS box complex formation wherever it is found, distinct interaction motifs may have evolved convergently in lineages where the PI motif is missing or altered, but higher order complexes still form (Lange et al., 2013). The double-flowered mutant phenotype of an ornamental cultivar of *Thalictrum thalictroides *(Ranunculaceae) may also be the result of disrupted PPIs between C and E class MADS box proteins (Galimba et al., 2012).

*APETALA3 (AP3*)-like and *PI*-like genes comprise the two main lineages of B class MADS box genes. In all core eudicots investigated thus far, B class proteins bind DNA as obligate heterodimers: AP3-like proteins cannot bind DNA without PI-like proteins and vice-versa (Riechmann et al., 1996a,b). The AP3–PI heterodimer in *Arabidopsis *goes on to autoregulate late *PI* and *AP3 *expression (Honma and Goto, 2000). This obligate heterodimer relationship is uncommon in the large MADS box gene family (Riechmann et al., 1996a), and obligate heterodimerization coupled with autoregulation is a rare, if not unique regulatory mechanism. All angiosperms investigated thus far have at least one *AP3*-like and one *PI*-like gene, and AP3-like and PI-like proteins bind DNA as obligate heterodimers in distantly related angiosperms, including the grass *Zea mays* (Vandenbussche et al., 2004; Whipple et al., 2004; Drea et al., 2007; Kramer et al., 2007). The only characterized B class proteins isolated from a gymnosperm thus far (the Gnetalean *Gnetum gnemon*) bind DNA as homodimers (Winter et al., 2002; Wang et al., 2010). These data, taken together, suggested that the obligate heterodimerization relationship evolved from a homodimerizing ancestor shortly after the duplication event that led to the *AP3* and *PI *gene lineages, and prior to the diversification of the angiosperms. However, PI homologs from *Lilium* were found to be capable of homodimerizing and heterodimerizing (Winter et al., 2002), but with no other data points, it was unclear whether this was an autapomorphy or indicative of a broader evolutionary trend. The single PI-like protein (J-PI) in *Joinvillea*, a close grass relative, can homodimerize (Whipple and Schmidt, 2006). PI-like homodimerization has also been observed in *Chloranthus* (Chloranthaceae; Li et al., 2005) and *Eschscholzia *(Lange et al., 2013). Together with the data from *Lilium*, these data imply the intriguing convergent evolution of obligate heterodimerization both in the monocots and in the lineage leading to the core eudicots. What remains to be deciphered is why obligate B class heterodimerization evolved at least twice. What, if any, is the functional difference between B class homodimers and heterodimers? One hypothesis suggests that the convergent evolution of obligate AP3–PI interaction is not the result of drift, but rather because the AP3–PI heterodimer confers a selective advantage: a robust switch in floral development (Lenser et al., 2009). It must be stated that all investigations into B class homo- vs. heterodimerization have been conducted *in vitro. *There is no evidence as of yet that PI-like homodimers function *in planta*.

The C class genes (*PLENA* and *FARINELLI*) of *Antirrhinum *have subfunctionalized, in part because of shifting PPIs. *PLENA* controls both male and female organ identity (stamens and carpels), while *FARINELLI* confers only male organ identity, both in *A. majus* and when overexpressed in *A. thaliana *flowers (Davies et al., 1999; Causier et al., 2005; Airoldi et al., 2010). When ectopically expressed, PLE, like AG, is capable of specifying both male (stamen) and female (carpel) organ identity, but FAR confers only stamen identity. This functional divergence has been traced to a single glutamine insertion in FAR, the result of an altered splice site. This amino acid insertion affects PPIs with the E class SEPALLATA (SEP) proteins: FAR can only interact with SEP3, while AG can interact with SEP1, 2, and 3. This change in PPIs, overlaid on *SEP* homolog expression patterns, has resulted in the subfunctionalization of *FAR* and *PLE*. Structural and regulatory changes have acted in concert to effect functional differentiation (Airoldi et al., 2010). In the genus *Medicago* (Fabaceae), a major difference in fruit morphology is correlated with a similar single amino acid insertion into SHATTERPROOF (SHP)-like MADS box proteins. Rather than disrupting PPIs, however, the amino acid insertion may strengthen the interaction between *Medicago *SHP and SEP3 homologs (Fourquin et al., 2013).

Outside of the MADS box genes, there is evidence that PPIs affect natural variation in altered trichome density (Symonds et al., 2011) and light response in *A. thaliana *(Filiault et al., 2008), domestication traits in wheat (Simons et al., 2006), and flowering time in barley (Turner et al., 2005). Trichome density, in particular, changes in response to herbivore pressure, and has a fitness effect (Mauricio, 1998). The bHLH transcription factor *ATMYC1* was found to underlie a QTL for trichome density in four separate *A. thaliana* mapping populations. A single amino acid change (P189A) was sufficient to abolish binding of atmyc1 to TTG (TRANSPARENT TESTA GLABRA) and GL1 (GLABROUS1) in yeast two hybrid assays (Symonds et al., 2011). Both TTG and GL1 are essential for trichome initiation in *A. thaliana* (reviewed in Balkunde et al., 2010). Presumably it is this altered interface with the trichome initiation pathway that results in reduced trichome initiation in plants with the L*er atmyc1* allele. In a cautionary tale for evolutionary biologists, positive selection acting on the *ATMYC1* coding sequence was detected, but the region under selection was downstream of the trichome-reducing P189A substitution (Symonds et al., 2011).

### COMPETITIVE INHIBITION AND DOMINANT NEGATIVES

Competitive inhibition of transcription factors by similar, but truncated, proteins represents one special PPI that has repeatedly surfaced as a regulatory mechanism (Staudt and Wenkel, 2010; Seo et al., 2011a). For example, the HD-ZIPIII transcription factor REVOLUTA, a key regulator of vegetative development (reviewed in Floyd et al., 2006), is negatively regulated by the LITTLE ZIPPER (ZPR) proteins. HD-ZIPIII transcription factors consist of four domains: a DNA-binding homeodomain, a leucine zipper domain, a START domain predicted to bind small hydrophobic molecules, and a MEKHLA domain (discussed below). All of the HD-ZIPIII proteins bind DNA as dimers. One class of genes that is upregulated by REV in particular is the *ZPR* genes. In contrast to the HD-ZIPIII proteins, the only recognizable domain in the ZPR proteins is the leucine zipper domain (Wenkel et al., 2007; Kim et al., 2008). The ZPR proteins bind REV *in vitro*, and inhibit DNA binding by REV. The ZPR overexpression phenotypes resemble those seen when HD-ZIPIII function is reduced. These data suggest a negative feedback loop, where the HD-ZIPIII proteins upregulate *ZPR* expression and the ZPR proteins repress HD-ZIPIII genes by sequestering them in inactive heterodimers. *ZPR *genes have been found in *Arabidopsis*, maize, and rice, so this form of gene regulation may be relatively ancient in the flowering plants (Wenkel et al., 2007).

The form of competitive inhibition demonstrated in the HD-ZIPIII/ZPR system is evident in a number of other transcription factor families: IDD14 in starch accumulation (Seo et al., 2011b), ZHD5 and MIF in floral and leaf development (Hong et al., 2011), Aux/IAA and ARF proteins in auxin response (Ulmasov et al., 1997;Vernoux et al., 2011), MEINOX and BELL proteins in leaf development (Magnani and Hake, 2008), and the MYB proteins DIVARICATA and RADIALIS in establishing floral symmetry (Corley et al., 2005; Raimundo et al., 2013). The smaller, competitive inhibitor proteins have been termed microProteins or short interfering peptides (siPEPs; Staudt and Wenkel, 2010; Seo et al., 2011a). Very few of these systems have been investigated in an evolutionary context, so it remains unclear whether the siPEPs have arisen because of convergent evolution, or whether they share a common ancestor with their competitors and have undergone domain loss. The second scenario, common ancestry and domain loss, seems more likely given the widespread occurrence of domain loss in gene family evolution (Bornberg-Bauer et al., 2010). In the case of IDD14, the competitive inhibitor is the result of an alternative splicing event, suggesting that there may be many more examples of competitive inhibition lurking in plant genomes (Staudt and Wenkel, 2010; Seo et al., 2011a).

The above examples of competitive inhibition are reminiscent of the effects of dominant-negative alleles. Often, dominant-negative alleles are thought to “poison” the protein complexes they are part of, ultimately causing a mutant phenotype. Two separate cases of dominant-negative alleles in natural variation have recently been described in *A. thaliana *and in *Helianthus annuus *(Asteraceae). In *A. thaliana*, QTL mapping of natural variation in branching pattern resulted in the identification of a naturally occurring allele of the MADS box protein AGL6 that, in combination with other loci, causes reduced shoot branching. This dominant-negative allele results in single amino acid replacement (P201L) in the C-terminus, a region of the protein thought to mediate higher-order PPIs (Huang et al., 2012).

In *H. annuus*, the sunflower, three tandem duplicate homologs of the *A. thaliana *floral inducer *FT* *(FLOWERING LOCUS T)* underlie a single large-effect QTL for flowering time. All three paralogs show divergent expression patterns, indicative of subfunctionalization. In addition, there is a frameshift mutation in the domesticated version of one of the paralogs, *HaFT1*, that causes a 17aa insertion in the encoded protein. In *A. thaliana*, the frameshift *HaFT1* allele abrogates the early flowering phenotype (under long days) conferred by a 35S::*HaFT4* transgene. This dominant-negative effect may result from disrupted PPIs between HaFT1 and its floral induction partners. The frameshifted allele is found almost exclusively in domesticated, not wild, sunflower cultivars, and there is evidence for a selective sweep at the genomic region surrounding *HaFT1*, indicating that this altered gene may have been a target of selection during domestication (Blackman et al., 2010).

## ALTERED DOMAIN CONTENT

Protein domains have been described that target proteins to particular cellular compartments [e.g., nuclear localization signals (Lange et al., 2007)]; that act as repressor or activator domains [e.g., the EAR repression domain (Ohta et al., 2001)]; that function in mediating the assembly of protein complexes [e.g., the PDZ domain (Kennedy, 1995)]; that act as post-translational modification (PTM) sites (Lusser et al., 2001); and that target proteins for destruction [e.g., the D box, (Ho et al., 2008)], to name a tiny subset of the existing diversity. The evolutionary origin of many characterized protein domains is often unclear or unexamined, except in a few cases. In a study of the evolution of plant protein domain gain and loss, Kersting et al. (2012) showed that new, plant-specific domains have emerged throughout plant history, but the highest rate of novel domain emergence was detected on the branch leading to the seed plants. This study also demonstrated that the arrangement of domains in individual proteins varies considerably, particularly at shallower phylogenetic levels. Lineage-specific domain architectures are not uncommon (Kersting et al., 2012).

Plant-specific gene lineages may possess domains present in all eukaryotes, but in land-plant-specific combinations (Xing et al., 2013). For example, the F-box and the tubulin DNA-binding domain are both found in all eukaryotes, but they are found adjacent to one another only in plants (Charoensawan et al., 2010). Similarly, HMG-box and AT-rich interaction domains are found in combination only in plants (Hansen et al., 2008). To catalog all characterized protein motifs and domains, and their occurrence in plant genomes, is beyond the scope of this paper. Instead, we have chosen to discuss examples where new functional domains in plant proteins have arisen through defined mechanisms, and to discuss examples where domain loss has been shown to have some defined functional consequence.

### NOVEL DOMAINS FROM HORIZONTAL GENE TRANSFER

There is evidence for horizontal gene transfer (HGT) between closely allied eukaryotic species (Bergthorsson et al., 2003, 2004; Richardson and Palmer, 2007; Xi et al., 2013), for massive chloroplast–nuclear gene transfer (Martin et al., 1998, 2002; Stegemann et al., 2003), and for inter-species chloroplast movement under stress (Stegemann and Bock, 2009; Stegemann et al., 2012). Combined, these data support the notion that new genes and new domains may arise in plant genomes through HGT. Two examples in particular highlight the recruitment of domains from HGT (the MEKHLA and the AP2 domains) to key developmental processes in plants.

The AP2 domain is found in 144 *Arabidopsis *transcription factors with diverse, important roles in plant development and in stress response (Okamuro et al., 1997). Outside of *Arabidopsis, *the AP2 domain has been found in all lineages of green plants investigated – from green algae to monocots. In *P. patens*, four proteins with AP2 domains have been found to be important for specifying cell-type identity (Aoyama et al., 2012). The AP2 domain was initially considered to be plant-specific (Riechmann and Meyerowitz, 1998), but more sophisticated database-searching methods revealed the existence of AP2 domains in homing endonucleases from a cyanobacterium (*Trichodesmium erythraeum*), a ciliate (*Tetrahymena thermophila*), and in two phages. No AP2 domains were detected in any other eukaryotes, apart from plants and *T. thermophila*.**The *T. erythraeum* AP2 domain aligns best with plant AP2 domains, and is also capable of binding DNA in a sequence-specific manner (Magnani et al., 2004).

Multiple lines of evidence support the hypothesis that the AP2 domain arose in plant genomes through HGT from a prokaryote, rather than convergent or divergent evolution:**(1) There is homology between the cyanobacterial gene and plant AP2-containing genes that extends beyond the AP2 domain. (2) Very few (15%) AP2/ERF transcription factor genes have introns. (3) The identified non-plant AP2 domains have a very similar predicted secondary structure to that of plant AP2 domains, and share more than 40% sequence identity with plant AP2 domains. (4) The nature of homing endonucleases themselves: homing endonuclease genes duplicate themselves in a process of gene conversion (Magnani et al., 2004). In addition, there is evidence that they have moved extensively, through HGT, into all of the biological kingdoms (reviewed in Stoddard, 2011).

The MEKHLA domain of REV is important for proper protein function (Prigge et al., 2005), but it is not required for transcriptional activation. Instead, the MEKHLA domain may be acting as a negative regulator of REV (Magnani and Barton, 2011). Phylogenetic analysis suggests that the MEKHLA domain, characteristic of HD-ZIP III transcription factors, found its way into plant genomes through either HGT from plant-associated bacteria, or through mass nuclear transfer from the early chloroplast (Martin et al., 2002; Mukherjee and Buerglin, 2006).

The evolution of the AP2 and MEKHLA domains demonstrates how new domains may arise and adopt important regulatory roles in plant development. Both domains were recruited into plant genomes at deep nodes in their phylogenetic histories: AP2 and MEKHLA domains are found in all plants, including the green alga *Chlamydomonas*. Given the hypothesized widespread occurrence of HGT in plant genomes (Richardson and Palmer, 2007), these examples may not be remarkable. Careful phylogenetic analysis, focused on particular domains rather than genes, may well reveal many more horizontally transferred protein domains.

### NOVEL DOMAINS FROM FRAMESHIFT MUTATIONS

The B class MADS box genes *AP3* and *PI *are key for controlling petal and stamen development in many flowering plants (Coen and Meyerowitz, 1991; Vandenbussche et al., 2004; Whipple et al., 2004; Drea et al., 2007; Kramer et al., 2007). There are two AP3-like genes in most core eudicots, products of a gene duplication event that generated the euAP3 and TM6 gene lineages (Kramer et al., 1999). The two gene lineages possess distinct, evolutionarily conserved C-terminal domains (Vandenbussche et al., 2003; Kramer et al., 2006). The derived euAP3 C-terminal domain (including the euAP3 motif) was probably generated through a frameshift mutation that occurred at the base of the core eudicots (Kramer et al., 2006). Where they have been investigated, the euAP3 and TM6 gene lineages have distinct but overlapping roles in floral development (Vandenbussche et al., 2004). There is some evidence that this functional distinction in the core eudicots is mediated, at least in part, by the proteins’ divergent C-termini (Lamb and Irish, 2003). Frameshift mutations have arisen and been maintained in other taxa with *AP3-*like gene duplications, and in other gene lineages, although the functional significance of the novel motifs generated has not been extensively investigated (Litt and Irish, 2003; Vandenbussche et al., 2003; Kramer et al., 2006; Pabón-Mora et al., 2012).

### DOMAIN LOSS

Domain loss can be detected by phylogenetic analysis of individual protein families (Zhang and Wang, 2005; Finet et al., 2013), and a large-scale analysis of protein domain evolution in plants revealed that domain loss occurs fairly frequently in plant lineages, particularly at family and subfamily-specific phylogenetic levels (Kersting et al., 2012). Although relatively easy to detect, the functional significance of these novel domain architectures is difficult to assess. Three examples where the function of domain loss has been shown involve the terpene synthase biosynthetic enzymes (Hillwig et al., 2011); the E class MADS box transcription factors from rice (Christensen and Malcomber, 2012); and a NAC domain transcription factor from *A. thaliana* (Li et al., 2011).

Plant terpene synthases are thought to have evolved from diterpene synthases, essential enzymes in the gibberellin synthesis pathway. Huge chemical diversity exists in plants, partly because of the evolution of the terpene synthases. Terpene synthases have lost the central γ-domain characteristic of diterpene synthases. There is some evidence that γ-domain loss has occurred multiple times in various taxonomic groups, but it remains uncertain whether γ-loss was a single evolutionary event, or the result of several parallel domain losses (Hillwig et al., 2011).

The E class MADS box genes of rice *Leafy hull sterile* (*LHS*) and *OsMADS5* (*OSM5*) are the products of a gene duplication event that occurred early on in the diversification of the grasses (Christensen and Malcomber, 2012). *Lhs1* mutants are characterized by leafy lemmas, paleas, and lodicules, fewer stamens, and occasional extra pistils and/or florets (Jeon et al., 2000). *osm5* mutants show a very mild floral phenotype: partial fusion between the lodicules (petal homologs) and the lemma and palea (sepal homologs; Agrawal et al., 2005). There is a premature stop codon in OSM5, shortly after the DNA-binding MADS domain of the protein. Perhaps because of this truncation, postdating the gene duplication event that produced *OSM5*, OSM5 has a different spectrum of binding partners to LHS, which may contribute to its divergent function (Cui et al., 2010; Christensen and Malcomber, 2012).

The Cvi and L*er* accessions of *Arabidopsis* have differing sensitivities to fructose. A QTL for fructose sensitivity was cloned, and it corresponds to a gain-of-function mutation in a NAC domain transcription factor gene (ANAC089). A premature stop codon in the Cvi allele leads to a truncated protein, missing a predicted membrane-bound domain (Li et al., 2011). In some NAC transcription factors, the membrane-bound domain serves to retain the protein in the cytoplasm in an inactive form (Seo et al., 2008). Without the membrane-anchoring domain, ANAC089 is constitutively active in the nucleus, probably as a transcriptional activator. Although it does demonstrate some of the molecular diversity that might be tolerated in nature, the Cvi allele of ANAC089 is rare, and possibly deleterious (Li et al., 2011).

## ALTERED ACTIVITY OF TRANSCRIPTIONAL REPRESSORS AND ACTIVATORS

FT (FLOWERING LOCUS T) and TFL (TERMINAL FLOWER) are distantly related paralogous regulators of flowering in *Arabidopsis*. FT is a floral integrator, and *FT* expression induces flowering. TFL is a floral repressor and maintains indeterminate growth of the shoot apical meristem. This functional distinction between FT and TFL has been separately traced to a single amino acid difference in the predicted anion-binding pocket (Y85 in FT and H88 in TFL; Hanzawa et al., 2005) and to differences in an external protein loop termed “segment B” (Ahn et al., 2006). There is evidence that FT and TFL exert their respective functions as part of transcriptional activator and repressor complexes (reviewed in Taoka et al., 2013). Y85 in FT and H88 in TFL may be working to recruit transcriptional coactivators or corepressors, either alone or in concert with “segment B” (Ahn et al., 2006; Taoka et al., 2013).

Similarly, two FT homologs in *Beta vulgaris* (sugarbeet) show antagonistic functions in the regulation of flowering. *BvFT2 *function is conserved with *FT* and acts as a floral promoter while *BvFT1* represses flowering. The antagonistic functions of BvFT1 and BvFT2 have been traced to differences at three amino acid residues in “segment B.” *BvFT1* and *BvFT2* appear to be the products of a relatively recent gene duplication event: *BvFT2* homologs have not been found outside of the genus *Beta *(Pin et al., 2010).**

Some soybean (*Glycine max, *Fabaceae) cultivars display a narrow leaflet phenotype, long been known to be controlled by a single gene, *ln. Ln* has been mapped to a genomic region that includes a single gene – *Gm-JAG1*– a homolog of the *A. thaliana *zinc-finger gene *JAGGED*. A single amino acid substitution (D9H) in the transcriptional repressor EAR motif of *Gm-JAG1* is likely to be the causal *ln *mutation, rendering *Gm-JAG1* non- or hypofunctional (Jeong et al., 2012). In addition to altering leaf morphology, the* ln *mutation affects the number of seeds per fruit (You et al., 1995; Dinkins et al., 2002). This example highlights how pleiotropic protein mutations may be tolerated and maintained in populations, possibly because of some fitness advantage. In this case, a fitness advantage may be conferred by the higher seed set of the *Ln/ln* heterozygote (Dinkins et al., 2002).

*Teosinte glume architecture1 (tga1)*, an SBP-domain transcription factor, has been identified as a key locus in the domestication of maize from its wild progenitor, teosinte (Wang et al., 2005). Morphological differences between maize and teosinte ears are probably caused by a single coding change (K6N) in *Tga1*. This single amino acid change alters the biochemical function of TGA1, but the exact mechanism of this change remains unclear (Preston et al., 2012). Given the degree of morphological change associated with this single amino acid change, it is reasonable to hypothesize that TGA1 is a transcriptional activator, activating the set of genes responsible for the development of teosinte-like glume and inflorescence morphology. The single amino acid change observed in maize was sufficient to abolish, or significantly alter, this role of TGA1 (Wang et al., 2005).

## ALTERED PROTEIN STABILITY

Protein degradation is one common mechanism of post-translational gene regulation. In plants, polyubiquitylation of proteins, followed by proteolysis mediated by the 26S proteasome, is a particularly prevalent mechanism of post-translational regulation (Vierstra, 2003). Examples of altered protein stability, possibly because of altered polyubiquitylation and degradation, have been observed in the light-sensing cryptochromes and phytochromes, known to be degraded in a light- and ubiquitin-dependent manner (El-Assal et al., 2001; Maloof et al., 2001; Filiault et al., 2008).

Light responses, such as flowering time, vary considerably amongst *A. thaliana *accessions (Maloof et al., 2001). Multiple independent inactivations of FRIGIDA and FLOWERING LOCUS C have been identified in the study of natural variation in flowering time (reviewed in Alonso-Blanco et al., 2009), but structural changes in light-sensing cryptochromes and phytochromes have also been implicated. For example, a novel allele of *CRYPTOCHROME-2 *(*CRY2*) underlies a large-effect QTL controlling daylength sensitivity (El-Assal et al., 2001). A single missense amino acid substitution in CRY2 (V367M) results in a more stable protein as compared to the more common L*er *allele (El-Assal et al., 2001). The same amino acid substitution in CRY2 (V367M) is also associated with shorter fruits, and decreased ovule number (El-Assal et al., 2004). A single amino acid (M548T) substitution in the phytochrome protein PHYA underlies reduced far-red light sensitivity in the Lm-2 accession of *A. thaliana* (Maloof et al., 2001). The substituted amino acid is able to affect multiple aspects of PHYA function: the photochemical properties of Lm-2 PHYA are affected by the M548T substitution; Lm-2 PHYA levels remained high in the light; and Lm-2 PHYA showed reduced autophosphorylation activity (Maloof et al., 2001). It is conceivable that the observed amino acid substitutions in both CRY2 and PHYA are interfering with some aspect of the phosphorylation, polyubiquitination, and 26S-mediated protein degradation pathway.

Hilscher et al. (2009) surveyed naturally occurring *A. thaliana* accessions for variation in trichome density. A single amino acid change, K19E, in the MYB domain transcription factor gene *ENHANCER OF TRY AND CPC 2* (*ETC2*), underlies one large effect trichome density QTL. K19, although highly conserved in single-repeat R3 MYB proteins, is not in a characterized protein domain, but may represent an ubiquitination site. In the low-density accessions, where this lysine is replaced with a glutamate, ubiquitination of the ETC2 repressor may have been reduced or lost, resulting in higher stability of ETC2 and, ultimately, fewer trichomes (Hilscher et al., 2009). An interesting point arising from this study is the relationship between trichomes and root hairs. ETC2 is the only characterized single-repeat R3 MYB gene family member that affects trichome density, but not root hair density. The K19E replacement, found at a relatively high frequency in naturally occurring accessions, may be tolerated because it occurs in a gene with low pleiotropy (Hilscher et al., 2009).

## HYPOMORPHIC AND HYPERMORPHIC ALLELES

Mutations that either decrease or increase protein function can be termed hypomorphs or hypermorphs, respectively (Muller, 1932). Examples of both hypomorphic and hypermorphic alleles in natural variation in a number of *A. thaliana *phenotypes have been described.

Hyperaccumulation and salt tolerance have repeatedly been associated with altered functionality of transporters and biosynthetic enzymes. Amino acid substitutions in conserved domains of HMA3 and HMA5 underlie *A. thaliana* QTL for Cd accumulation (Chao et al., 2012) and Cu tolerance (Kobayashi et al., 2008), respectively. The amino acid substitutions in HMA3 result in a hypofunctional translocator and, ultimately, higher Cd accumulation. Similarly, high sulfate accumulation in the Shahdara accession of *A. thaliana* (Loudet et al., 2007) and differences in salt tolerance between rice accessions (Ren et al., 2005) have been separately associated with hypomorphic alleles.

The late flowering Sy-0 accession of *A. thaliana *is distinctive in its morphology. The basal rosette is enlarged, aerial rosettes form in the axils of stem leaves, and early floral meristems revert to indeterminate growth (Poduska et al., 2003). A single amino acid replacement in the pre-mRNA processing factor, HUA2, is responsible for the majority of the Sy-0 aerial rosette phenotype. HUA2 has been shown to positively regulate the flowering genes *AG* (floral patterning, floral determinacy) and *FLC *(flowering time). In the Sy-0 accession, *AG* function is attenuated, and *FLC* expression is enhanced. Thus, the single Sy-0 amino acid replacement in HUA2 (K525E) is a partial loss-of-function (hypomorphic) allele with respect to its effects on *AG*, and a gain-of-function (hypermorphic) allele with respect to *FLC* expression. Although the morphological phenotype exhibited by the Sy-0 accession is not rare, the nucleotide polymorphism that causes the K525E amino acid replacement is rare. In a survey of 113 *A. thaliana *accessions, only Sy-0 was found to possess the causative single nucleotide polymorphism (SNP; Wang et al., 2007).

Naturally occurring accessions of *A. thaliana* exhibit considerable diversity in the rate of leaf production. One accession, Est-1, shows both slower leaf production, as well as extensive necrosis on older leaves. Both slower leaf production and late onset leaf necrosis in Est-1 are due to gain of function (hypermorphic) mutations in a single gene, *ACCELERATED CELL DEATH6 (ACD6). ACD6* encodes a transmembrane protein involved in the regulation of salicylic acid accumulation and the defense response. The increased activity of ACD6 observed in Est-1, and 14 other *A. thaliana *accessions, may confer enhanced pathogen resistance, but with costs. Enhanced pathogen resistance comes at the price of reduced biomass (fewer, smaller leaves), which in turn is associated with fitness costs (Abreu and Munné-Bosch, 2009; Todesco et al., 2010).

## MICRO- vs. MACROEVOLUTIONARY DYNAMICS IN PROTEIN EVOLUTION

We have divided our discussion into six broad categories of protein change, but we could also have divided the examples according to the evolutionary scale at which the change was predicted to occur (**Table 1**). Evolutionary change can be considered microevolutionary (occurring within a single population or species) or macroevolutionary (transcending species boundaries; Gould, 2002). When protein evolution is considered with these categories in mind, do certain changes occur preferentially on a micro- or macroevolutionary scale? It must be stated that all evolutionary events probably happen at a microevolutionary scale, within a population, but the scale at which we observe these events changes. Some categories of change were detected at both micro- and macroevolutionary scales, including active site evolution of enzymes, altered activity as a transcriptional activator or a repressor, and the evolution of PPIs. The evolution of competitive inhibition appears to occur primarily on macroevolutionary time scales, while dominant negatives were detected exclusively at a microevolutionary scale. Dominant-negative alleles and competitive inhibition are similar in character, and it is conceivable that dominant-negative alleles might represent the first step on one pathway to the evolution of competitive inhibition. Domain loss, observed at both micro- and macroevolutionary scales, may represent another pathway leading to competitive inhibition.

The existing examples of DNA-binding domain evolution occur on very deep, macroevolutionary time scales. Similarly, there were no examples of novel domains at microevolutionary timescales. Are these events so rare, and so often deleterious, that they are seldom uncovered in the study of population-level natural variation? Or, would systematic analysis of DNA-binding or protein domain architecture at a population-level reveal microevolutionary examples?

At the opposite side of the spectrum, but similarly illuminating, lie changes that were detected predominantly on microevolutionary scales. In addition to dominant negatives, hypo- and hypermorphic alleles and altered protein stability were detected almost exclusively on microevolutionary, or intrageneric, time scales. These examples may suggest where to look for innovation on macroevolutionary scales. These changes, sometimes causing drastically altered phenotypes, are tolerated in natural environments. Often it is difficult to distinguish functional and phylogenetic signal from the noise in evolutionary analyses of gene families. Perhaps looking for altered stability of evolutionary variants, for example, might yield insight into the functional consequences of molecular evolution. Altered protein stability, in particular, may represent one way in which a protein’s function might stay intact, but the protein may persist for a shorter or longer period of time. This could conceivably result in a heterochronic shift (Klingenberg, 1998) in a particular trait.

## CONCLUSIONS

One interesting point arising from our survey of the existing literature is that proteins can change in a number of ways that were not uncovered here. One class of changes, in particular, that remained elusive was PTMs. The examples of altered protein stability may have ultimately been because of altered PTMs, but that remains to be determined. The absence of altered PTMs in the study of protein evolution is perhaps because many of the PTMs of individual extant proteins are still incompletely understood, so assessing PTMs in an evolutionary context remains extremely challenging. In the case of QTL cloning, PTM alterations may not be tolerated very often, and will therefore vary only very rarely on microevolutionary scales. Examples do arise in mutant analyses (Soppe et al., 2000; Kim et al., 2006), so more cases of natural variation in PTMs may be forthcoming. PTMs have clearly arisen and diversified in proteins and the study of their evolution represents an interesting area of future exploration.

Although many of the discussed changes primarily affect transcription factors, the phenotypic outcomes of these changes are often vastly different. Even within one class of change, altered PPIs, one altered interaction affects trichome density in *Arabidopsis*, another affects floral morphology in *Thalictrum*. Although similar biochemical changes might have occurred, the ultimate phenotypes on which natural selection might act are distinct and not evolutionarily equivalent.

Genetic analysis (QTL cloning) has deepened our understanding of the molecular underpinnings of phenotypic diversity to a considerable degree. As more QTL are uncovered and cloned, no doubt this understanding will grow ever deeper. But systematically cloning QTLs will not tell us everything there is to know about the evolution of plant form and function. It remains important to combine all of the strategies available to us, including phylogenetic analyses of gene families, structural analyses, and functional analyses of proteins in an evolutionary context, in order to gain a more complete picture of protein evolution. It would also be extremely informative to know how many of the QTL that have been cloned confer adaptive phenotypes, or have the potential to be adaptive under certain conditions. Although challenging, field and laboratory selection tests on some of the more promising accessions would no doubt yield fascinating results.

## Conflict of Interest Statement

The authors declare that the research was conducted in the absence of any commercial or financial relationships that could be construed as a potential conflict of interest.
